# Sarcoidosis Resembling Angiokeratomas: A Case Report

**DOI:** 10.7759/cureus.56322

**Published:** 2024-03-17

**Authors:** Alexandra L McLennan, Clay J Cockerell, Vicky Z Ren

**Affiliations:** 1 Dermatology, Baylor College of Medicine, Houston, USA; 2 Dermatopathology, Cockerell Dermatopathology, Dallas, USA

**Keywords:** skin, sarcoidosis, non-caseating, lung, hilar, granuloma, cutaneous, angiokeratoma

## Abstract

Sarcoidosis, a multifaceted systemic disorder characterized histologically by the presence of non-caseating granulomas, has a wide array of cutaneous manifestations. We describe a case of a 74-year-old woman with a complex medical history, who presented with asymptomatic hyperpigmented papules on her lower extremities. Histological examination of a punch biopsy specimen showed nodular and angiocentric patterns of granulomatous inflammation consistent with sarcoidosis, and chest radiography demonstrated bilateral hilar opacities, supporting the diagnosis. To our knowledge, this specific cutaneous presentation of sarcoidosis has not been described before, and it can easily be mistaken for other conditions. Therefore, this case underscores the importance of recognizing atypical cutaneous morphologies of sarcoidosis, particularly in patients with complex medical histories, to facilitate accurate diagnosis and timely intervention. We aim to increase awareness among clinicians regarding the diverse manifestations of sarcoidosis, thereby enhancing diagnostic acumen and patient care.

## Introduction

Sarcoidosis, a systemic inflammatory condition characterized by non-caseating granulomas, manifests in various organs, including the lungs, lymph nodes, skin, heart, central nervous system, and gastrointestinal tract, with an annual prevalence ranging from 2.3 to 11 per 100,000 individuals [1,2]. This multifaceted disorder affects diverse demographic groups, with Black individuals and those of Scandinavian descent exhibiting notably higher prevalence rates compared to others [1,3]. Cutaneous involvement, observed in a significant proportion of patients, presents with a diverse array of manifestations, ranging from the more well-recognized erythema nodosum and lupus pernio to atypical presentations such as verrucous lesions and necrotic ulcers [4,5]. When sarcoidosis is not suspected, it can be misdiagnosed as psoriasis, granuloma annulare, cellulitis, acne, or lichen planus, among numerous other benign and malignant conditions [6]. Here, we present the case of a 74-year-old woman, who presented with atypical cutaneous lesions resembling angiokeratomas, which were incidentally diagnosed as sarcoidosis upon biopsy. Recognizing these atypical manifestations is crucial for accurate diagnosis, timely treatment initiation, and furthering our understanding of sarcoidosis pathology.

## Case presentation

**Figure 1:**
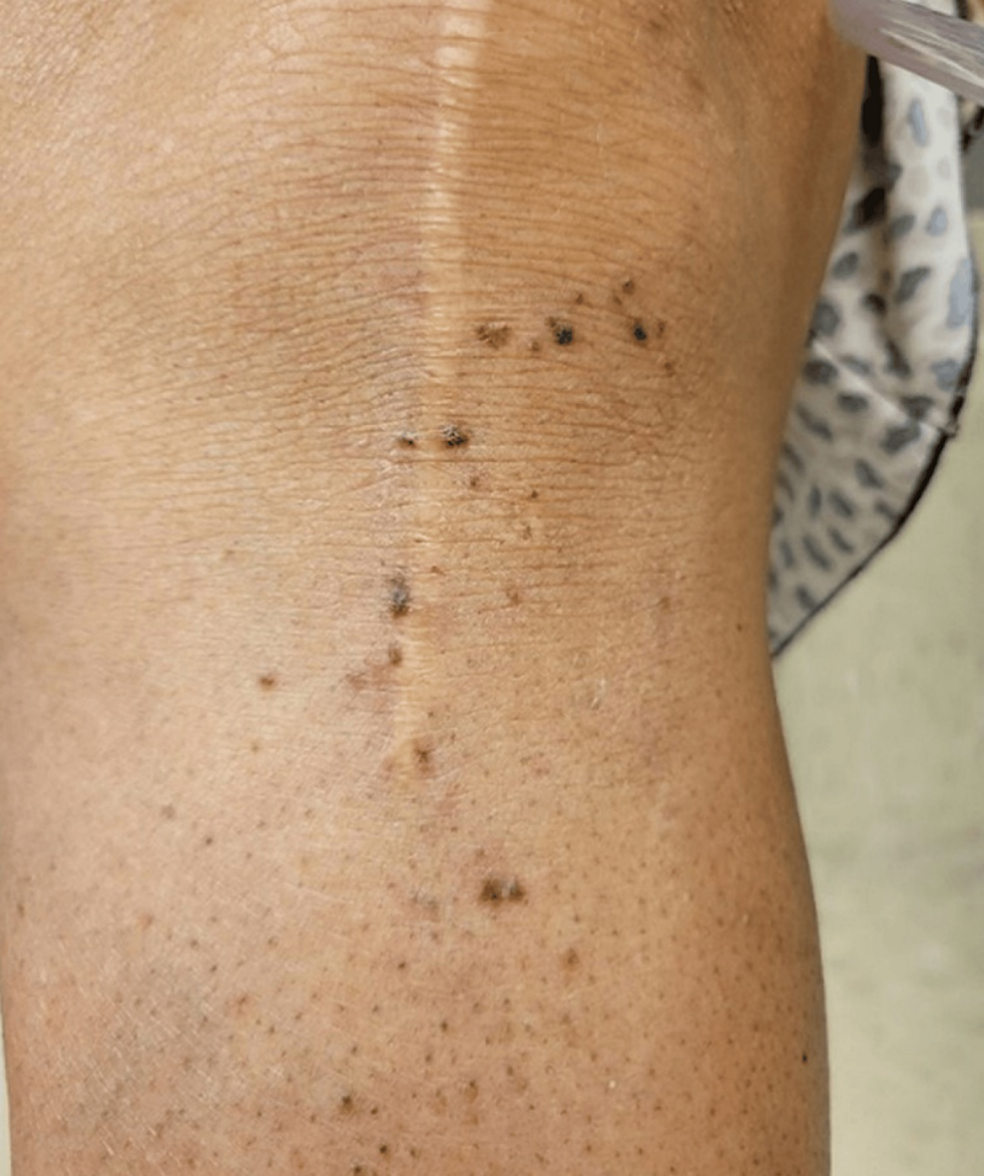
Several grouped, follicular, dark brown papules on the anterior shin

**Figure 2:**
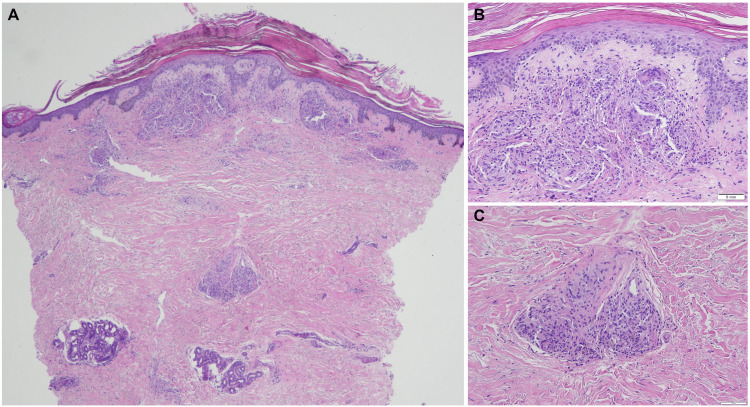
Histopathology of the punch biopsy (A) Punch biopsy of the right shin demonstrating dermal fibrosis and non-caseating granulomas in the superficial and deep dermis (hematoxylin & eosin, x4). (B) Discrete, non-caseating granulomas composed of epithelioid histiocytes with abundant eosinophilic cytoplasm, and macrophages surrounded by lymphocytes and rare multinucleated giant cells (hematoxylin & eosin, x20), and (C) perivascular lymphocytic infiltration (hematoxylin & eosin, x20)

A 74-year-old Black female with hypertension, obstructive sleep apnea, chronic obstructive pulmonary disease (COPD) requiring 3 L/minute of continuous oxygen via nasal cannula, diastolic heart failure, type 2 diabetes mellitus, and stage 3a chronic kidney disease presented to a dermatology clinic with a one-year history of asymptomatic, hyperpigmented papules on her lower extremities. These papules appeared approximately one year after initiating losartan, which was subsequently discontinued by cardiology. Upon physical examination, follicular hyperpigmented papules were observed on the anterior shins, resembling angiokeratomas (Figure 1); however, their follicular base raised suspicion for conditions such as lichen planus or discoid lupus erythematosus. The emergence of these new lesions prompted concern for the patient, particularly as they persisted despite discontinuation of losartan and showed minimal improvement with urea cream and other over-the-counter emollients. This led to a decision to obtain a punch biopsy from the right leg for histological examination to aid in diagnosis (Figure 2).

**Figure 3:**
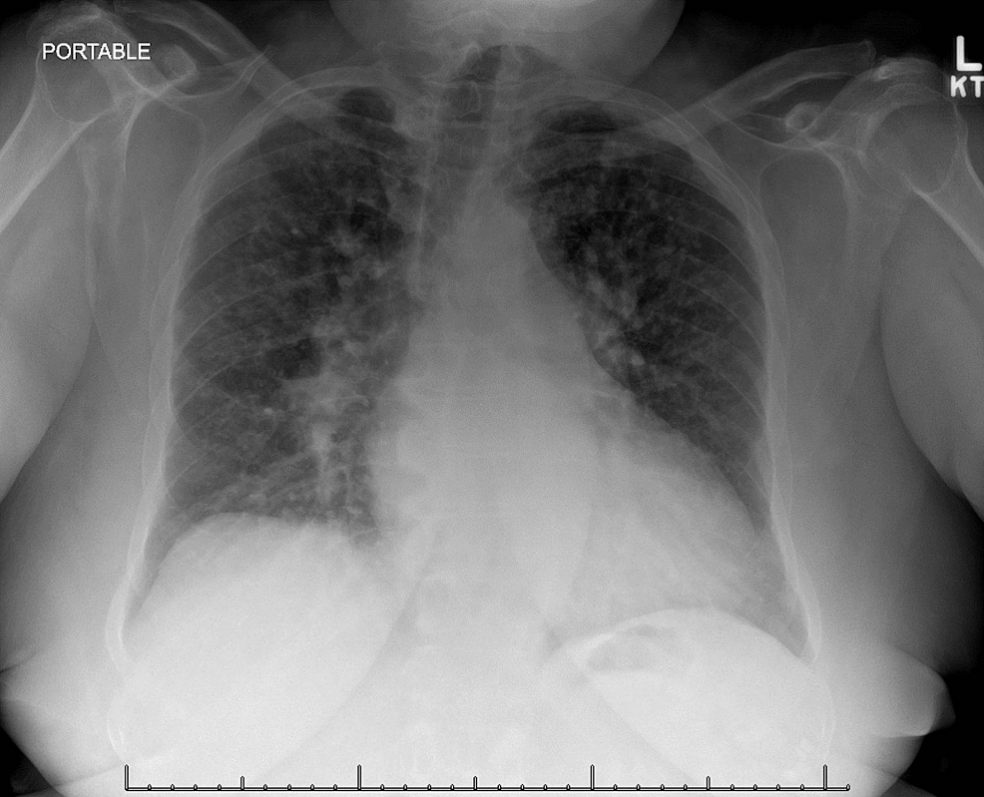
Chest radiograph showing reticulonodular opacities in both lung fields

Histological examination revealed non-caseating granulomas with a sparse marginal lymphocytic infiltrate in the superficial and deep dermis. Within the surrounding fibrosis, islands of epithelioid cells containing few Langhans giant cells were observed. Based on these findings, a diagnosis of sarcoidosis was made. Subsequent chest radiography depicted bilateral hilar opacities, further supporting the diagnosis (Figure 3). The patient was prescribed clobetasol 0.05% ointment for cutaneous sarcoid and advised to follow up with her specialty physicians, particularly pulmonology and cardiology.

**Figure 4:**
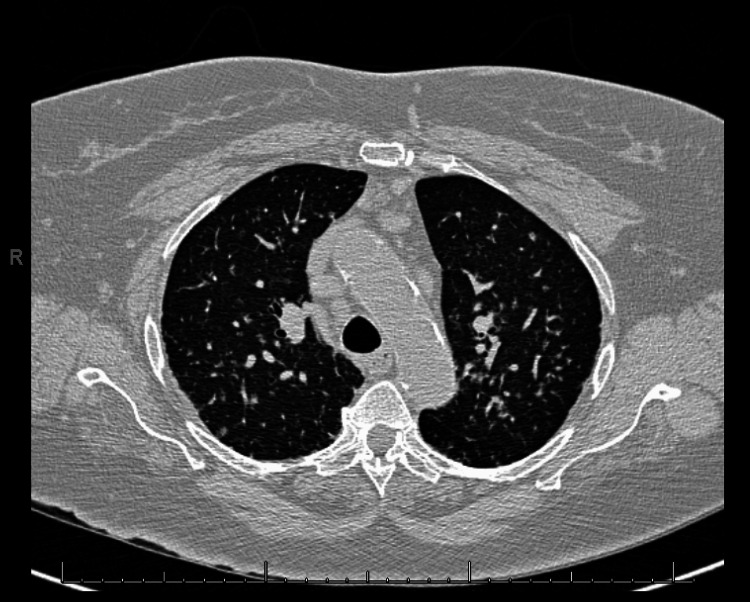
High-resolution CT imaging of the chest revealing numerous bilateral pulmonary nodules accompanied by extensive mediastinal and hilar lymphadenopathy

Given her comorbidities, the patient had been followed by pulmonology for presumed COPD for over 10 years, receiving treatment with inhalers. Sarcoidosis had not been considered during this time. Prior to the development of any cutaneous findings, she experienced shortness of breath and multiple syncopal episodes. Changes detected via echocardiography consistent with developing heart failure were noted, yet sarcoidosis was not initially suspected. In light of the results of the skin biopsy and the patient's deteriorating cardiopulmonary symptoms, repeat echocardiography, continuous electrocardiography, and cardiac magnetic resonance imaging revealed signs of heart failure but no evidence of infiltrative disease consistent with cardiac sarcoidosis. As suggested by radiographic imaging, a chest computed tomography scan conducted for the first time in five years showed the presence of nodules that were not previously observed in imaging studies, consistent with progressive pulmonary sarcoidosis (Figure 4). Consequently, the patient was started on oral azathioprine 50 mg daily before she was hospitalized for acute-on-chronic respiratory failure, upon which intravenous steroids were initiated and tapered to oral prednisone on discharge. At follow-up with pulmonology, oral methotrexate 10 mg weekly was initiated in place of azathioprine and steroids for maintenance therapy of systemic sarcoidosis.

## Discussion

With an annual incidence ranging from 2.3 to 11 per 100,000 individuals, sarcoidosis is a complex systemic disorder characterized by the formation of granulomas in various organs such as the lungs, lymph nodes, skin, heart, central nervous system, and gastrointestinal tract [1,2]. This condition demonstrates a wide-ranging impact across diverse demographic groups [3]. While its onset can occur irrespective of ethnicity or age, Black individuals and those of Scandinavian descent exhibit a notably higher prevalence compared to other populations [1]. Typically, sarcoidosis presents in adults under the age of 50, with a peak incidence between 25 and 40 years of age; additionally, a secondary peak is noted among women over 50 years old, as in our patient [7]. Despite its prevalence and potential severity, sarcoidosis receives less attention regarding its dermatological manifestations compared to pulmonary involvement in current literature [8]. However, given the skin's accessibility for clinical assessment, it represents a critical domain for unraveling the varied morphology, pathogenesis, and management of sarcoidosis.

The etiology of sarcoidosis remains enigmatic, with hypotheses suggesting a chronic immunological reaction in genetically susceptible individuals exposed to unidentified external antigens [4,8]. This pathophysiology is characterized by granulomatous inflammation driven by a Th1-mediated immune response, where antigen-presenting cells initiate CD4+ T cell activation via major histocompatibility complex presentation, leading to subsequent secretion of pro-inflammatory cytokines [9,10]. Clinically, sarcoidosis presents with a broad spectrum of cutaneous manifestations, often posing a diagnostic challenge due to its potential mimicry of other dermatological conditions. While erythema nodosum, lupus pernio, and maculopapular or plaque-like eruptions are well-documented, newer observations continually expand our understanding of the disease [4]. Uncommon presentations, such as atrophic lesions, psoriasiform plaques, and papulonodular eruptions, along with rare manifestations, like angiolupoid, hypopigmented, and alopecic sarcoidosis, highlight the heterogeneous nature of cutaneous sarcoidosis [4,5,11,12]. In certain cases, the skin may play a predominant role in disease manifestation and treatment decisions, while in others, it may serve as an incidental or secondary concern, albeit providing a convenient site for diagnostic biopsy confirmation.

In this case report, we delineate cutaneous manifestations of sarcoidosis that, to the best of our knowledge, have not been previously described. Among the reported cutaneous variations, our patient's presentation aligns most closely with the maculopapular type, albeit with notable deviations. Typically, maculopapular sarcoidosis presents as small, red-brown to purple papules less than 1 cm in diameter that may combine to form annular lesions [2,13]. However, our patient's lesions manifested as several discrete and grouped, follicular, dark brown papules on the extremities, lacking the characteristic translucent yellow-brown tint against an erythematous background and umbilicated centers seen in the classic maculopapular type [2,14].

There are many potential therapies for cutaneous sarcoidosis, though no one therapy is universally effective [5]. Localized involvement typically warrants the use of topical or intralesional steroids, with the latter administered at intervals of two to three weeks for enhanced efficacy [6]. Systemic glucocorticoids and alternative agents, such as methotrexate or azathioprine, are reserved for those with internal organ involvement or cutaneous lesions that are extensive, advancing, or cause functional impairment [15].

## Conclusions

Sarcoidosis is a complex systemic disorder with diverse cutaneous manifestations. Despite extensive characterization of many skin findings, the identification of novel presentations, as exemplified in this case report, emphasizes the ongoing necessity for vigilance and awareness among clinicians. By recognizing and documenting these atypical dermatological features, healthcare providers can improve diagnostic accuracy, optimize patient management, and contribute to the growing body of knowledge surrounding this enigmatic disease.
